# Incorporation of engineered nanoparticles of biochar and fly ash against bacterial leaf spot of pepper

**DOI:** 10.1038/s41598-022-10795-8

**Published:** 2022-05-20

**Authors:** Zill-e-Huma Aftab, Waqar Aslam, Arusa Aftab, Adnan Noor Shah, Adnan Akhter, Usama Fakhar, Iffat Siddiqui, Waseem Ahmed, Farzana Majid, Jacek Wróbel, Muhammad Danish Ali, Muzammil Aftab, Mohamed A. A. Ahmed, Hazem M. kalaji, Asad Abbas, Umar Khalid

**Affiliations:** 1 Department of Plant Pathology, University of the Punjab, Lahore, 54590 Pakistan; 2 Department of Physics, University of the Punjab, Lahore, Pakistan; 3 Eastern Cereal and Oil Seed Research Centre, Ottawa, Canada; 4 Department of Botany, Lahore College for Women University, Lahore, Pakistan; 5 Department of Physics, Government College University Lahore, Lahore, Pakistan; 6 NUCES-Fast University Lahore Campus, Lahore, Pakistan; 7 Punjab University College of Pharmacy, University of the Punjab, Lahore, Pakistan; 8 Department of Horticulture, The University of Haripur, Hatter Road, 22620 Pakistan; 9 Plant Production Department (Horticulture—Medicinal and Aromatic Plants), Faculty of Agriculture (Saba Basha), Alexandria University, Alexandria, 21531 Egypt; 10 Department of Plant Physiology, Institute of Biology, Warsaw, University of Life Sciences SGGW, Now-oursynowska 159, 02-776 Warsaw, Poland; 11 Department of Bioengineering, West Pomeranian University of Technology in Szczecin, 17 Słowackiego Street, 71-434 Szczecin, Poland; 12 School of Horticulture, Anhui Agricultural University, Hefei, 230036 China; 13 Department of Agricultural Engineering, Khwaja Fareed University of Engineering and Information Technology, Rahim Yar Khan, Punjab, 64200 Pakistan

**Keywords:** Plant sciences, Pathogenesis

## Abstract

In agriculture, the search for higher net profit is the main challenge in the economy of the producers and nano biochar attracts increasing interest in recent years due to its unique environmental behavior and increasing the productivity of plants by inducing resistance against phytopathogens. The effect of rice straw biochar and fly ash nanoparticles (RSBNPs and FNPs, respectively) in combination with compost soil on bacterial leaf spot of pepper caused by *Xanthomonas* *campestris* pv. *vesicatoria* was investigated both in vitro and in vivo. The application of nanoparticles as soil amendment significantly improved the chili pepper plant growth. However, RSBNPs were more effective in enhancing the above and belowground plant biomass production. Moreover, both RSBNPs and FNPs, significantly reduced (30.5 and 22.5%, respectively), while RSBNPs had shown in vitro growth inhibition of *X.* *campestris* pv. *vesicatoria* by more than 50%. The X-ray diffractometry of RSBNPs and FNPs highlighted the unique composition of nano forms which possibly contributed in enhancing the plant defence against invading *X.* *campestris* pv. *vesicatoria*. Based on our findings, it is suggested that biochar and fly ash nanoparticles can be used for reclaiming the problem soil and enhance crop productivity depending upon the nature of the soil and the pathosystem under investigation.

## Introduction

Capsicum or bell pepper or sweet pepper (*Capsicum* *annum* L.) is a crop of Solanaceae family and genus ‘capsicum’. These medium-sized fruit pods have wonderful colors (green, red, orange and yellow) thick and brittle skin with a glossy outer cover and a fleshy texture. It is a highly appreciated crop being good source of vitamin A, C, E, thiamine, beta carotene, folic acid and vitamin B6 and has great therapeutic values^1,2^. In Pakistan, the area under pepper has been 62,742 hectares in 2018–2019 with a total production of 145,856 tonnes and comes at 5th position worldwide. Bacterial leaf spot (BLS) caused by *Xanthomonas* *campestris* pv. *vesicatoria* results in severe damage to sweet pepper. The bacterium attacks leave, fruits, and stems causing blemishes on these plant parts. It is a gram-negative, rod-shaped bacterium that can survive in seeds and plant debris from one season to another^3–5^. The pathogen can devastate a pepper crop by early defoliation of infected leaves and disfiguring fruit. In severe cases, complete crop failure has occurred due to this disease. Marketable yield is reduced both by defoliation and damaged fruits^2,6^. For the management of BLS different techniques have been under application such as chemical control^7^, cultural methods^8,9^, biocontrol strategies^10^, and use of resistant plant genome^11^.

In recent years, organic amendment, including crop residues, compost, organic waste and biochar application has become an auspicious strategy for the control of soil-borne diseases because of its strengths as, cost-effectiveness, resource utilization and environmental protection^12–14^. Biochar (BC) or black gold is a novel organic soil amendment with some special physical and chemical properties that has been increasingly discussed in agriculture as a strategy for the sequestration of recalcitrant carbon into soils to increase soil fertility^15^, improve plant growth and suppression of soil-borne diseases^16–18^. Additionally biochar has been proven as an effective suppressor of plant diseases caused either by soil-born or air-born bacterial or fungal plant pathogens^18,19^.

Fly ash has been defined as the fine particulate by-product released into the atmosphere together with gases as a result of combustion processes^20^. The dynamic physic-chemical properties (low bulk density (1.01–1.43 g cm^−3^), hydraulic conductivity and specific gravity (1.6–3.1 g cm^−3^), while the moisture retention ranging from 6.1% at 15 bar to 13.4% at 1/3 bar and being rich in P, K, Ca, Mg and S and micronutrients like Fe, Mn, Zn, Cu, Co, Ba, Mo, Cd and Ni)^21,22^ of fly ash make it a potential source in agricultural applications, as improving biological and physic-chemical properties of soil^23,24^ as competent as the compost and Biochar. Currently, Fly ash has also shown significant inhibitory effects on root-knot nematodes in carrot and soybean plants as well as control of some bacterial populations^25–28^.

The incorporation of engineered nanoparticles has gained undeniable importance in our daily life from electronics to medicine and agriculture. In agriculture, for instance, nano-pesticides, nano-fertilizers and nano-sensors are in direct applications to agricultural soils to get enhanced crop productivity and reduce cost^29^, or control plant pathogens^30,31^. Characterized nanoparticles of Fly ash^32,33^ and Biochar^34^ have been extensively used in the agriculture sector not only to reduce the hazards of deposited chemical pesticides and fertilizers but also to control infectious pathogens and to improve crop yields^35–37^.

In this study we explored the synthesis of nanoparticles from rice straw biochar (RSBNPs) and fly ash (FNPs) and secondly the potential of prepared nanoparticles was assessed against bacterial leaf spot of pepper caused by *Xanthomonas* *campestris* pv. *vesicatoria*.

## Materials and methods

### Experimental site

The experiment was carried out at the experimental station of the Department of Plant Pathology, (31º 29′ 42.2664″ N, 74º 17′ 49.1316″ E, 217 m altitude) Faculty of Agricultural Sciences, University of the Punjab, Lahore, Pakistan, from March 2019 to April 2021. The local climate is semi-arid (Köppen climate classification BSh) with an average temperature of 40 °C and 350 mm annual rainfall and rainy season July–September.

### Plant material and soil substrate

*Capsicum* *annum* L. seeds (Yolo Wonder) were purchased from the local seed market (Ghula Mandi, Lahore) and surface sterilized with 70% ethanol for 10 min followed by washing with 50% NaOHCl solution (100 mL of NaOHCl + 100 mL of distilled + 50 µL tween-20 detergent) and thrice washing with distilled deionized water^38^. These seeds were sown in clay pots ¾ filled with sterilized 20% leaf compost soil (The soil texture was a sandy loam (82.88% sand, 13.04% silt, and 4.08% clay) with a pH of 7.88 and an electrical conductivity (EC) of 1.55 dS/m (measured using a pH meter and an EC meter); organic matter content (OM) of 0.54%; containing 3% total N and 1.5% total C; having a C/N ratio of 0.5; and containing 12, 68, and 100 mg·kg^−1^ of Ca, P, and K, respectively). Fully developed plants at 4–5 leaf stage were transplanted, into clay pots of bigger size (Volume: 2 L, 15.5 cm height × 14 cm width)^83,84^ with the same soil composition up to 1–2 inches depth with 2–3 plants per pot. Lighter irrigations were applied on a day-to-day basis to keep the water level at about 60%^39^. Then established young plants in pots were transferred to open areas where seedlings were exposed to light so that they can carry their photosynthetic activity^17^.

### *Xanthomonas campestris *pv*. vesicatoria* culture acquisition

Pure culture of *Xanthomonas* *campestris* pv*.* *vesicatoria* (FCBP-DNA B0003) was acquired from First Fungal culture Bank of Pakistan (FCBP), Faculty of Agricultural Sciences, University of the Punjab, Lahore, Pakistan. The inoculum was prepared by re-culturing in LB broth (MERCK, USA) based on Lennox formulation and incubating on a shaker at 120 rpm for 36 h at 28 ± 2 °C. Bacterial culture was then centrifuged at 5,000 rpm for 10 min at 4 °C. The suspension was diluted through serial dilution process to obtain the bacterial concentration of 10^8^ at 600 nm wavelength having an optical density of 0.3 in the spectrophotometer^40^.

### Biochar and nanoparticles production

TLUD (Top-Lit UpDraft) portable kiln method^41^ on-farm biochar production was used, with minor adjustments, to prepare biochar from Rice Straws collected from field areas of University of the Punjab, new campus, Lahore, at pyrolysis temperature of 500 °C to be used in this experiment. Fly ash was procured from the textile industry as leftover after burning corn cobs and coal as fuel (usually is a micro-scale ultrafine particulate with a size below 100 µm). Physico-chemical properties of rice straw biochar and fly ash were determined^42,85,86^ before use for further nanoparticles production are summarized in Tables 1 and 2.

**Table 1:** Physico-chemical characterization of rice straw biochar.

Parameter measured	Value
pH	9.3
Basic gps (meq/g)	7.8
Acidic gps (meq/g)	1.8
Ash%	50
Density (g/cm^3^)	0.28
Surface area (*S* _*BET*_ ) (m^2^/g)	100
C wt%	53
H wt%	3.0
N wt%	1.5
O	42.4
C/N	25
H/C	0.08
O/C	0.79
**Alkaline elements (ppm (ug/g) by dry weight)**	
K	14,000
Mg	3500
Na	2190
Ca	7354
**Other essential elements mg/Kg**	
Fe	5754
P	2765
**Heavy toxic elements mg/Kg**	
Zn	0.01
Mn	575
Al	4231
Cu	4
Cd	0.05
Pb	0.62
Hg	0.93

**Table 2:** Physico-chemical characterization of fly ash.

Parameter measured	Value
Ash %	46
pH	9.75
EC dSm^−1^	2.43
C%	39.3
N (g Kg^−1^)	6.71
P (g Kg^−1^)	2.97
K (g Kg^−1^)	0.31
Ca (g Kg^−1^)	2.51
Mg (g Kg^−1^)	1.37
S (g Kg^−1^)	7.52

The nanocomposite of rice straw biochar (RSBNPs) and fly ash (FNPs) were isolated from their bulk materials following protocols of Yeu et al., 2019; Guo et al.^43,44^, by grinding bulk biochar into a commercial blender to produce fine biochar powder. Fly ash obtained was already packed in sealed polythene bags. Fly ash (30 g) and fine biochar powder were mixed in 800 mL of sterile water, separately. Both the solutions were shaken vigorously and autoclaved to physically and thermally disperse the bulk forms of biochar fine powder and fly ash. After the dispersion of bulk material, prepared solutions were passed through a 500 µm filter membrane to remove large particles. Filtrates were centrifuged twice at 3500 rpm for 25 min to isolate the nanoparticles in supernatant based on a density gradient. XRD, FTIR, analysis of both Biochar and fly ash nanoparticles and EDX of only biochar was done by following Du et al.^45^, from the Department of Physics, Lahore College for Women University, Lahore, Pakistan.

Biochar and fly ash nanoparticles were applied through drenching (Imada et al.)^46^ to chili plants by applying 50 mL of solution, containing nanoparticles (RSBNPs and FNPs), in the root zone by injecting with the help of a disposable syringe (Telemart: 10 cc, Bd).

### Chili pepper plant inoculation and disease assessment

The plants were grown for 7–8 days before the inoculation of the pathogen (*X.* *campestris* pv. *vesicatoria*). Leaves of chili plant were injured by the needle prick method of bacterial inoculation^47^. In this method, 8–10 clean needles were tightly held by a rubber band at equal heights. These needles were used to damage the leaves. Slight gentle injuries were done to leaves to provide entry sites to bacteria. Then the bacterial suspension was sprayed with the help of an atomizer. Inoculated plants were covered again with polythene bags and water was sprinkled on the inner bag surface to maintain high relative humidity.

The whole research trail was comprised of two groups. Each group was further divided into three treatments, having five replicates. Group I: Inoculated set: (1. Fly-ash nanoparticles + Xnth;, 2. Rice Straw Biochar Nanoparticles + Xnth; 3. Only Soil + Xnth). Group II: Un-inoculated set/Control group: (1. Fly-ash nanoparticles, 2. Rice Straw Biochar Nanoparticles, 3. Only Soil).

After the application of nanoparticles and inoculation of the pathogen, agronomic data were recorded as shoot and root length, weight^48^. Disease incidence was calculated by the following formula

$${\text{Disease}}\text{ incidence (\%) = }\frac{\text{Number of diseased plants}}{\text{Total number of plants}}\times 100.$$

Disease severity was calculated by formulating a disease grading scale in which severity was rated from 0 to 4 grades with zero indicating minimal or no disease symptoms to grade four showing 76% or above leaf area infected^49^.

### Consent for publications

All authors have read the manuscript and agreed for publishing it.

### Consent for plants/seeds

The authors declare that during the research work all national and local legislations have been followed before and after conducting the experiment and no rules have been violated during the whole experiment keeping the crop respect in consideration.

## In vitro *X. campestris *pv. *vesicatoria* and other isolated potential bacterial and fungal pathogens growth inhibition assay

The antimicrobial activity of RSBNPs and FNPs was investigated against *X.* *campestris* pv. *vesicatoria* used in this experiment. Agar well diffusion method^50^ was employed for the estimation of antimicrobial potential of RSBNPs and FNPs.

The antimicrobial activity of RSBNPs and fly FNPs was also investigated against microflora isolated from soil used in this experiment. Fungi and bacteria were isolated through serial dilution method on Malt Extract Agar (MEA) and Luria Bertani Agar (LBA), respectively. Agar well diffusion method^50^ was employed for estimation of antimicrobial potential of RSBNPs and FNPs against isolated fungal and bacterial isolates including *Escherichia* *coli*, *Erwinia* *spp***,** *Pseudomonas* *syringae,* *Xanthomonas* *campestris* pv. *citri***,** *X.* *campestris* pv. **v***esicatoria,* *Fusarium* *solani***,** *F.* *oxysporum,* *Alternaria* *alternata* and *Alternaria* *solani***,.**

### Statistical analysis

The experimental data were analysed by ‘Statistix version 8.1’ analytical software by analysis of variance (ANOVA), while the means were differentiated by Tuckey’s HSD test at P = 0.05. Additionally, the percentage data were transformed for disease incidence, severity and in vitro bacterial growth inhibition before analysis.

## Results

### Effect of rice straw biochar nanoparticles and fly ash nanoparticles on plant growth

Maximum shoot length (28 cm) was observed in un-inoculated rice straw biochar nanoparticles (RSBNPs) treated plants. In the pathogen inoculated set of treatments, maximum shoot length was observed in both fly ash and biochar-based nanoparticles treated plants (Table 3). While minimum shoot length of 8 cm was observed in pathogen inoculated control plants grown in soil only. RSBNPs had significantly enhanced root length as suggested by the results because maximum root length i.e. 27 cm was observed in uninoculated plants treated with RSBNPs. Pathogen inoculated plants grown in soil, RSBNPs + Soil and FNPs + soil had root lengths of 6.1 cm, 13.4 cm, and 11 cm, respectively. RSBNPs treated plants resisted the pathogen stress and had 101%, while, FNPs treated plants had shown a 65.1% increase in root length as compared to plants grown in only soil.

**Table 3:** Effect of rice straw biochar nanoparticles (RSBNPs) and fly ash nanoparticles (FNPs) and *Xanthomonas* *campestris* pv*.* *vesicatoria* inoculation on chili plant growth parameters including shoot length, root length as well as root and shoot weights.

Treatments	Shoot length (cm)	Shoot weight (g)	Root length (cm)	Root weight (g)
Only soil	13.2 ± 1.20^d^	0.47 ± 0.01^e^	6.66 ± 0.53^e^	0.285 ± 0.05f
Soil + Xnth	11 ± 1.00^de^	0.22 ± 0.03f.	6.1 ± 0.74^e^	0.1542 ± 0.03^e^
Soil + RSBNPs	25 ± 1.14^a^	2.09 ± 0.09^a^	22.8 ± 1.32^a^	1.9852 ± 0.33^a^
Soil + Xnth + RSBNPs	19.6 ± 0.51^c^	1.07 ± 0.15^c^	13.4 ± 1.21^c^	0.7144 ± 0.10^c^
Soil + FNPs	23.2 ± 1.07^ab^	1.73 ± 0.12^b^	21 ± 0.55^ab^	1.4172 ± 0.14^b^
Soil + Xnth + FNPs	19 ± 0.65^c^	0.75 ± 0.11^d^	11 ± 1.00^ cd^	0.456 ± 0.08^d^

Data represent mean values ± standard error and abcd denote significance levels.

With the addition of composite nanoforms derived from rice straw biochar and fly ash a significant increase in chili shoots weight was observed as compared to plants grown in only soil in both inoculated and un-inoculated sets of treatments (Table 3). Shoot weight was significantly increased in RSBNPs treated plants in both pathogen-inoculated and uninoculated chili plants. But highest average shoot weight was recorded in un-inoculated, RSBNPs treated plants as 2.039 g. In pathogen inoculated plants, the average shoot weight in only soil-grown plants was 0.219 g as compared to 1.067 g of RSBNPs treated and 0.748 g of FNPs treated plants.

An increase in root weight was observed in RSBNPs and FNPs treated plants. Very robust root hair growth was found in nanoparticles treated plants. In pathogen inoculated plants, average root weights were 0.154 g in soil-grown plants, 0.714 g in RSBNPs treated plants, and 0.456 g in FNPs treated plants. RSBNPs treated, pathogen inoculated plants had 150.7% more root weight as compared to un-inoculated, only soil grown plants and 363.3% more average root weight as compared to pathogen inoculated plants grown in only soil. On the other hand, FNPs treated, pathogen inoculated plants had 60% enhanced root weight as compared to un-inoculated only soil-grown plants and 195.7% more root weight in comparison with pathogen inoculated plants grown in only soil.

### Disease incidence and severity

**Figure 1:**
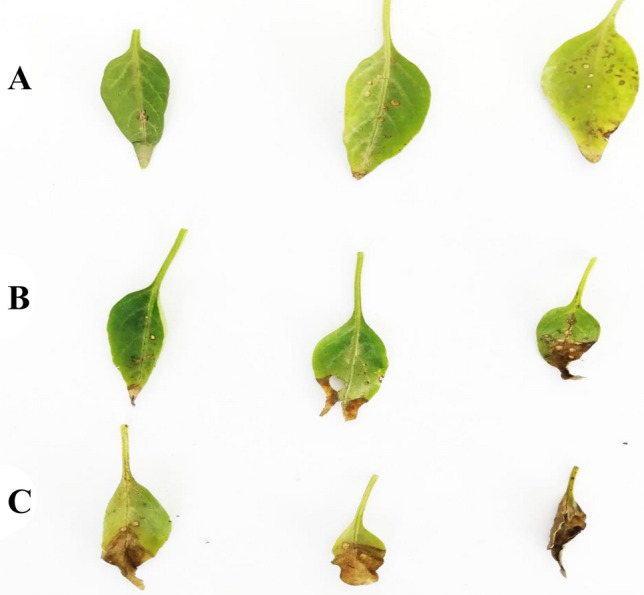
Leaves of chili plants showing disease symptoms in RSBNPs treated plants (**A**), FNPs treated plants (**B**) only soil-grown plants (**C**). In vitro inhibitory effect of rice straw biochar nanoparticles (RSBNPs) and fly ash nanoparticles (FNPs) on *Xanthomonas* *campestris* pv*.* *vesicatoria.*

Among the inoculated set of treatments, RSBNPs treated plants showed a different response to pathogen inoculation by showing significantly reduced disease incidence and disease severity (50 and 22.5%, respectively) as shown in Table 4. While there was a disease incidence of 100% in plants grown in only soil. The disease severity of FNPs treated plants was (30.5%) followed by the highest (94.5%) in plants grown in untreated soil. The severity of the disease symptoms on chili plant leaves treated with nanoparticles (RSBNPs, FNPs) and without any nanoparticles are shown in Fig. 1.

**Table 4:** Effect of rice straw biochar nanoparticles (RSBNPs) and fly ash nanoparticles (FNPs) on the incidence and severity of *Xanthomonas* *campestris* pv*.* *vesicatoria* in chili plants.

Treatments	Disease incidence (%)	Disease severity (%)	Disease rating category
RSBNPs + soil	50^c^	22.5 ± 2.84^c^	1
FNPs + soil	60^b^	30.5 ± 3.75^b^	1
Only soil	100^a^	94.5 ± 10.58^a^	4

In vitro inhibitory effect of RSBNPs and fly ash nanoparticles was evaluated against bacterial leaf spot caused by *Xanthomonas* *campestris* pv*.* *vesicatoria*. The zone of inhibition were calculated and shown in Table 4. RSBNPs have shown 51.2% growth inhibition of *Xanthomonas* *campestris* pv. *vesicatoria.* However, FNPs had shown inhibition of only 42.4% as compared to un-amended control. In addition to that both RSBNPs and FNPs had shown significant growth inhibition of isolated bacterial and fungal pathogens as summarized in Table 5.

**Table 5:** In vitro percentage (%) growth inhibition of five different phytopathogenic bacteria and four fungal isolates by RSBNPs and FNPs.

Bacterial pathogens	Percentage (%) growth inhibition	
	RSBNPs	FNPs
*Escherichia* *coli*	56.25 ± 8.45^ cd^	53.1 ± 5.21^ cd^
*Erwinia* *spp*.	52.5 ± 4.89^ cd^	65 ± 7.29^ab^
*Pseudomonas* *syringae*	73.1 ± 8.60^a^	64.6 ± 5.55^ab^
*Xanthomonas* *campestris* pv. *citri*	75 ± 7.84^a^	62.4 ± 6.74^ab^
*X.* *campestris* pv. *vesicatoria*	51.2 ± 6.67^ cd^	42.4 ± 3.94^e^
**Fungal pathogens**		
*Fusarium* *solani*	62 ± 6.83^ab^	60.2 ± 4.77^ cd^
*Fusarium* *oxysporum*	47.5 ± 3.99^e^	69.3 ± 3.01^ab^
*Alternaria* *alternata*	70 ± 5.50^ab^	58 ± 4.85^bc^
*Alternaria* *solani*	59.7 ± 6.36^ab^	52.5 ± 5.01^bcd^

### X-ray diffractometry of rice straw biochar nanoparticles and fly ash

**Figure 2:**
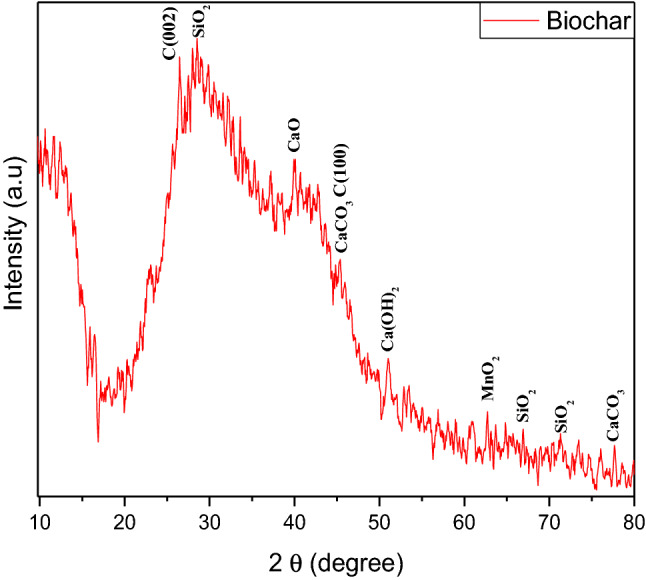
XRD spectrum of rice straw biochar.

XRD data of biochar is shown in Fig. 2. The range of the XRD spectrum is 2θ = 10–80°. In Fig. 2 different peaks are observed at various angles due to different elemental compositions. In the region of 20 to 30, a hump is observed due to C (002). Around 42–46°, another hump is observed due to C (100) which is attributed to condensed carbonized planes. In the XRD spectra there are three peaks which are observed around 28, 68, and 73 due to the concentration of SiO_2_ and well-matched with (JCPDS card no. 46-1045). A peak is detected around 39 due to CaO presence (JCPDS card no. 011-1160). A detected peak of Ca(OH)_2_ is well-matched with (JCPDS card no. 01-073-5492) around 51°. The presence of CaCO_3_ is detected at around 45 and 79° and confirmed through (JCPDS card no. 05-0586). Whereas a peak of MnO_2_ is well-matched with (JCPDS card no. 44-0141) and detected around 65°.

**Figure 3:**
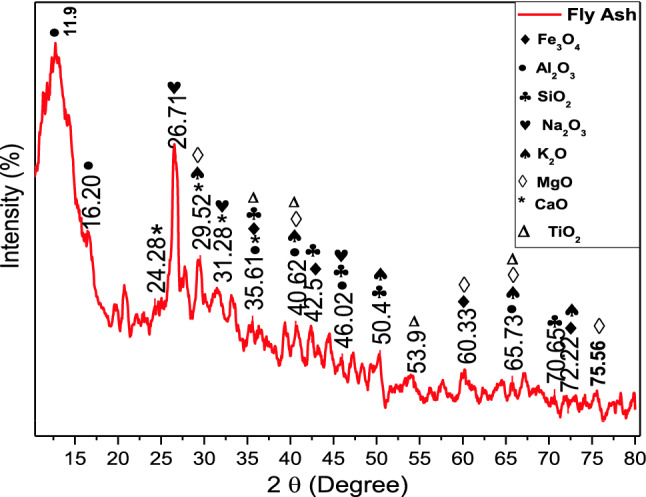
XRD spectrum of fly ash.

By using XRD the peak identification and material confirmation of the fly ash have been characterized and demonstrated, in the range 10–80 as shown in Fig. 3. The following graph showed that the material contained an appropriate amount of SiO_2_, Al_2_O_3_, TiO_2_, Na_2_O_3_, magnetite, K_2_O, MgO, and CaO. It can be observed that calcium, silica, and aluminium are the main elements of the fly ash and comprise 72% of the total mass of fly ash. In the XRD section the magnetite peaks are observed at 35.61, 42.5, 60.33 and 72.22°, which are well-matched with JCPDS card no. 73-2143, while the Al_2_O_3_ peaks were observed at 11.9,16.20, 35.61, 40.62, 46.02 and 65.73°, and according to JCPDS card no. 51-0769. The JCPDS card no. 80-2157 is matched with SiO_2_ and peaks are observed at 35.61, 42.5, 46.02, 50.4 and 70.65° while the Na_2_O_3_ is observed at the peaks of 26.71, 31.28, 46.02°. Due to the presence of K_2_O the JCPDS card no. 23-0493 is well matched at 29.52, 40.62, 50.4, 65.73 and 72.22°. The presence of MgO is detected on 29.52, 40.62, 60.33, 65.73, 75.66° and matched with JCPDS card no. 30-0794. The peaks of CaO found fit with JCPDS Card no. 28-0775 at peaks of 24.28, 29.52, 31.28, 35.61°. The other component TiO_2_ is well-matched with JCPDS card no. 33-1381 and the peaks were recorded at 35.61, 40.62, 53.9, 65.73°.

### Fourier Transformed Infrared Spectroscopy (FTIR)

**Figure 4:**
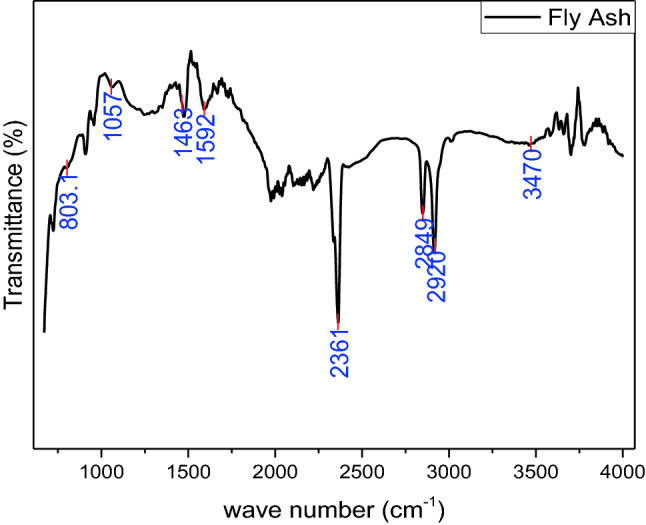
FTIR spectrum of fly ash.

FTIR spectrum of Fly ash is depicted in Fig. 4. Various peaks of fly ash due to different chemical bonding are noticed at 803.1, 1057, 1463, 1592, 2361, 2849, 2920, and 3470 cm^−1^. Due to O–H stretching peak of water bonding, an extreme is detected at 3470 cm^−1^^51^. Because of methylene and carbon symmetric and asymmetric stretching vibration, the peaks 2849 and 2920 are depicted in spectra. By the deformation of H–O–H bonding the vibration peaks were detected at 2361 and 1592 cm^−1^^52^. A peak of CO deformation was observed at 1463 cm^−1^^52^. By symmetric stretching of Si–O a small peak is detected at 1057 cm^−1^^52^. Due to out-of-plane C–H stretching a peak is depicted at 803.1 cm^−1^ and this is because of the presence of mullite^52^.

**Figure 5:**
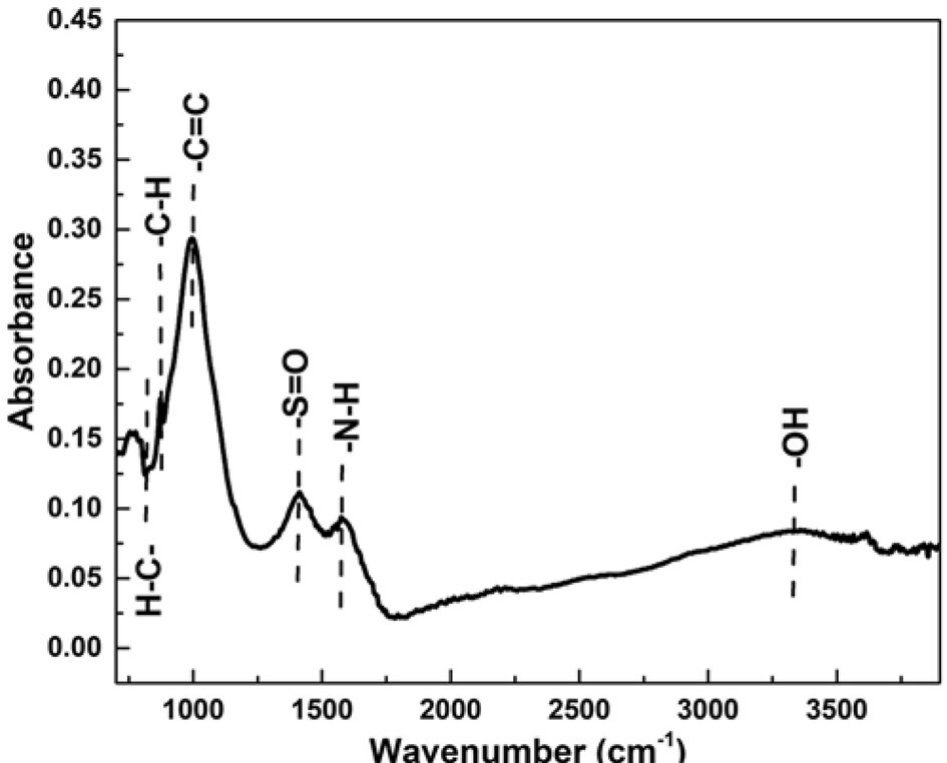
FTIR spectrum of biochar.

The FTIR spectra of Biochar against absorbance spectra are figured out in Fig. 5. Due to different chemical bonding structures, there are different peaks are observed in FTIR spectrum of biochar. An extreme O–H stretching vibration is detected at 3358.42 cm^−1^^53^. While the peaks of 1412.23 and 1575 cm^−1^ represent the presence of amine and sulphate groups, and the peaks are observed due to O=S stretching vibration in sulphate and N–H bending vibration in the amine group respectively^54^. A bond of C=C exhibits its presence with the help of a peak at 996.34 cm^−1^. Different peaks of C–H bending in the aromatic rings are observed in a range of 700 to 900 cm^−1^. A single C–H is observed at 872.62 cm^−1^ and a H–C bending peak of aromatic ring is noticed at 774.86 cm^−1^^54^.

### Scanning electron microscopy (SEM)

**Figure 6:**
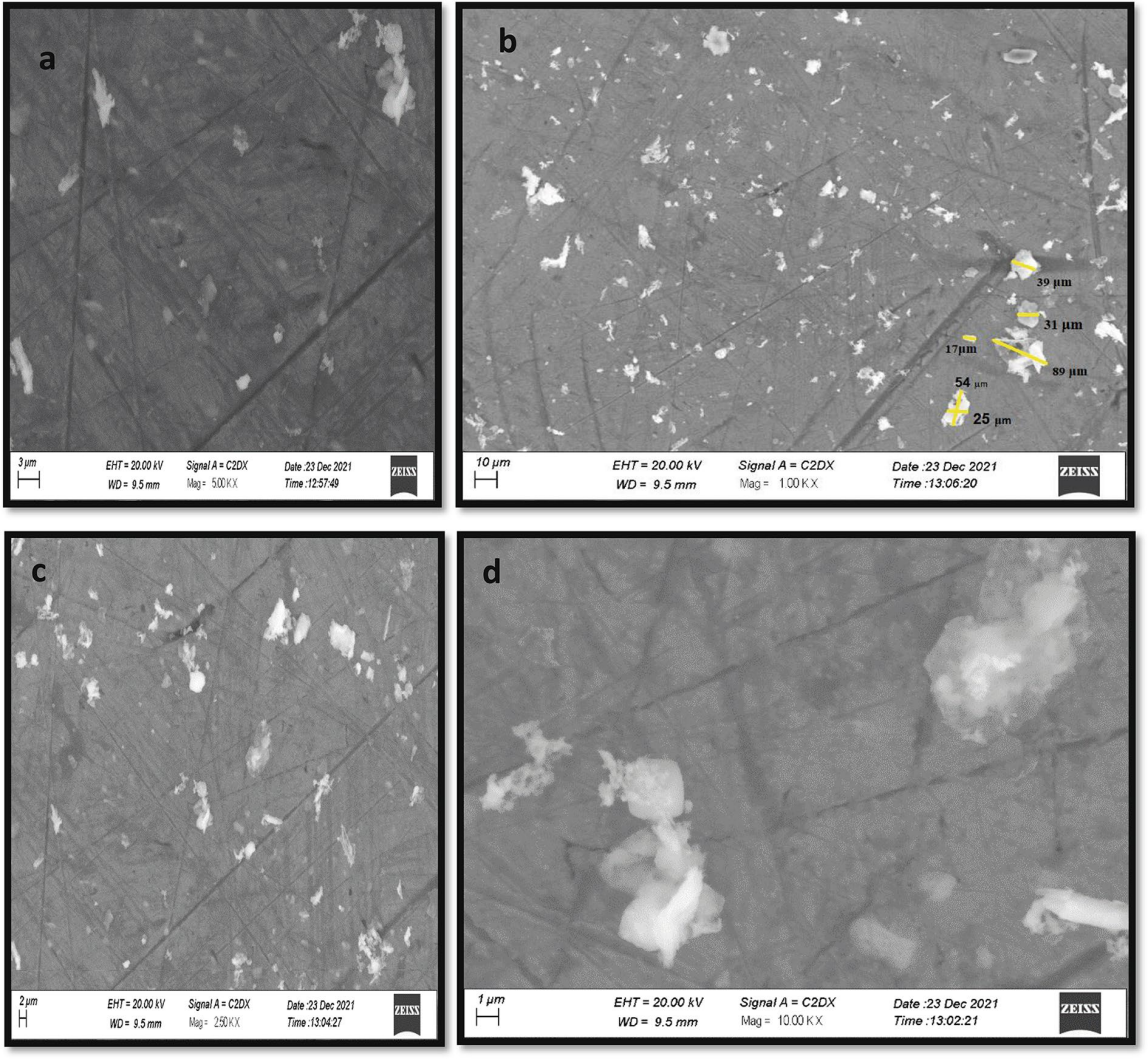
SEM images of fly ash.

The morphology of the Fly ash particles is studied through SEM. The SEM results are captured at 1,2,3, and 10 μm scale in Fig. 6a–d. The results of SEM indicate the irregular size distribution of the Fly ash particles. It is suggested that silica is responsible for the irregular shape of the particles^55^. Ceno-spheres, smaller spheres and irregularly shaped spheres are observed in Fly ash SEM morphology. The size of the particles is in between the range of 10 to 90 μm.

**Figure 7:**
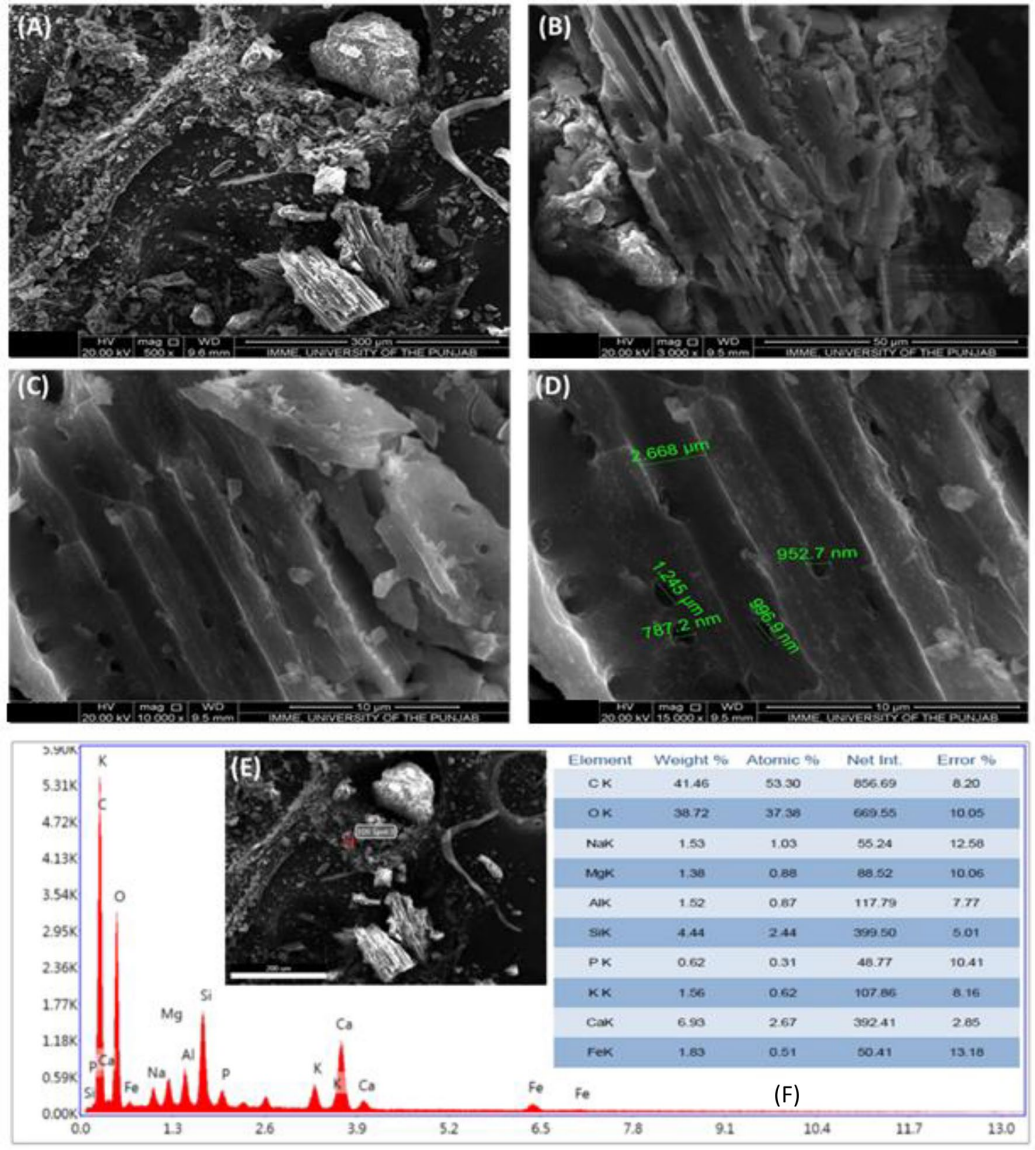
SEM images of biochar.

The morphology of the Biochar was analysed through Scanning electron microscopy (SEM) in Fig. 7. SEM micrographs were taken at four magnifications of 50 μm, 300 μm, 10 μm and 15 μm. It can be seen that the image at 50 μm showed a shattered pelletized structure and a tabular structure was obtained at 300 μm magnifications. Furthermore, at 10 μm of magnification, it can be seen that from the figure the biochar tabular pores are pored with specific particles on both sides of the biochar, while, on the other hand, the 15 μm magnification showed an obvious channel size of 2.668 μm and pores of 787.2 nm, 952.7 nm, 996.9 nm, and 1.245 μm (Fig. 7A–D).

The SEM–EDX or elemental analyses of cow manure biochar revealed a rich amount of mineral elements. A high amount of C contents was measured followed by O, Na, Mg, Al, Si, P, K, Ca and Fe (Fig. 7F).

## Discussion

The rise in unprecedented climatic changes like temperature and changing weather patterns had worsened the situation over the past few decades. While annual crop losses due to insect pests and diseases are estimated to range from 20 to 40% of total agricultural produce worldwide further escalating the hostility to existing food insecurity^56^. The discovery of innovative technological advancements in the agriculture sector is mandatory, to supersede an otherwise deteriorating global food scenario, in a sustainable manner. The recent innovations in scientific research, particularly, the advent of molecular nanotechnology have provided a ray of hope against all the odds through its effective role in drug delivery, target specificity, diagnostics, anti-microbial activity in the pharmacology and medicine industry^57^. Nanotechnology has marked its footprints in the field of agricultural research by its utility in establishing disease and pest diagnostic systems, phytohormonal delivery systems, nano-barcoding, enhancing germination of seeds, providing nano-vector for successful transfer of genes, establishing efficient and targeted slow-releasing chemical pesticides^58^.

Rangaraj et al.^59^ has reported that silica NPs as effective agents for building resistance against *Fusarium* *oxysporum* and *Aspergillus* *niger* in maize. Nanotechnology is being widely used in plant pathological studies^60^. There exist thorough studies on the effects of biochar in controlling plant diseases^61^. But major portion of studies on biochar involves a macro fraction of biochar and material behaves differently when used in nano (10^–9^) size in contrast to their bulk/macro forms. The present study was designed to fulfil the research needs on nano fractions of biochar and their role in controlling plant disease. Yue et al.^43^ attributed the increase in plant growth in response to biochar NPs to negating the effect of allelopathic materials in soil^43^. In accordance with our results, Xu et al.^62^ demonstrated that nano-biochar possess a unique set of physical and chemical traits other than in their bulk forms which enhanced root growth^63^. High, surface reactive tendencies and capacities to disperse allow them to attach and interact easily to root surfaces which is quite beneficial for protection of roots by physical means against heavy metal adversities.

Moreover, the nano biochar due to its smaller particle size has high mobility in soils and helps to transport water^64,65^. Bashir et al.^66^ used composts and ZnO-nanoparticles to evaluate their effect on growth parameters like the dry weight of roots, shoots, husk, and kernels, plant height and spike length of the plant concerned^67^. Results obtained suggested a strong effect of used nanoparticles and compost material on growth promotion. Furthermore, increased photosynthetic activity owing to nanoparticles inoculation, which reduces the effects of osmotic and oxidative stress, is well documented as the process increases the plant biomass^67–69^.

The decrease in disease incidence and severity can be attributed to, up-regulation of the innate immune response of plants against pathogens, due to the induction of nanoparticles^70^. Chandra et al*.*^71^ reported sufficient enhancement in plant’s response through activation of innate immunity by induction of chitosan nanoparticles which, in return, increased the activity of defense-related enzymes. Enhancement in total phenolic compounds, anti-oxidative enzymes and genes involved in defense mechanism was also reported due to the treatment of carbon base chitosan nanoparticles. Studies indicate that induction of carbon-based nanoparticles stimulate the production of enzymes related to defense mechanisms, like Peroxidase (PO), Phenyl Alanine ammonia Lyase (PAL), Poly Phenol Oxidase (PPO), and plant defense regulating molecules such as beta 1–3 glucanase, nitric oxide (NO) and etc. Nitric oxide is involved in many physiological processes^72^ including the regulation of the defense process in plants^73^.

Disease incidence and severity percentages of the bacterial pathogen were decreased which can be due to direct destructive effects of nanoparticles on the bacterial membrane as nanoforms of materials are electro-statically active and interact with the lipo-polysaccarhide structure of the bacterial membrane. As, XRD, FTIR and SEM data revealed novel characteristics of BNPs including azimuthal and parallel orientation of aromaticity, partly carbonized lamellae^74^. The hump around 42–46° due to C (100) proves a large amount of carbon present in the sample and due to this carbon presence, a crystalline orientation appeared simple in the form of peaks^75–77^, While in the case of fly ash NPs, the XRD, FTIR and SEM data is the evidence of the presence of different phases of Al and fly ash particles^78^.

Secondly, nanoparticles constituting, mostly, heavy metals bind with DNA/RNA molecules of bacteria by passing through the cell membrane and hinder transcription- translation process thus inhibiting bacterial proliferation^79^. Nanoparticles trigger the production of salicylic acid (SA), a phytohormone that activates the SAR mechanism in plants^80^. Carbon based nanoparticles triggered a systemic acquired resistance mechanism that provides resistance to infection to remote plant tissue from the site of its production^81^.

Fly ash is known previously^82^, to limit the papaya leaf curl disease spread along with the regulation of the vector population (*Bemisia* *tabaci*). However, there are also risks associated with fly ash use including leaching of heavy metals or changes in the microbial composition of the soil. So, caution should be practiced while using fly ash for agricultural purposes^86^.

Owing to increasing food demand, rapidly changing climate, high pathogens adaptability to climatic changes and hazardous effects of least efficient chemical control measures, the need for natural, effective, climate-friendly way of disease control is inevitable.

The NPs induced changes were significant regarding chilies growth and bacterial leaf spot suppression. However, the plant response to NPs was dependent on the source or material used for their production. RSBNPs could provide a better alternative to unchecked bulk use of pesticides. There is a need to check the possible hazards like dose, toxicological issues and eco-acceptability of these nanoforms. Further exploration of NPs utility obtained from fly ash and biochar would certainly help in managing plant diseases and addressing environmental concerns associated with toxic pesticides.

## Conclusion

In agriculture, the search for higher net profit is the main challenge in the economy of the producers and nano biochar attracts increasing interest in recent years due to its unique environmental behaviour and increasing the productivity of plants by inducing resistance against phytopathogens. The effect of rice straw biochar and fly ash nanoparticles (RSBNPs and FNPs, respectively) in combination with compost soil on bacterial leaf spot of pepper caused by *Xanthomonas* *campestris* pv. *vesicatoria* was investigated both in vitro and in vivo. The application of nanoparticles as soil amendment significantly improved the chili pepper plant growth. However, RSBNPs were more effective in enhancing the above and belowground plant biomass production. Moreover, both RSBNPs and FNPs, significantly reduced (30.5 and 22.5%, respectively), while RSBNPs had shown in vitro growth inhibition of *X.* *campestris* pv. *vesicatoria* by more than 50%. The X-ray diffractometry of RSBNPs and FNPs highlighted the unique composition of nanoforms which possibly contributed to enhance the plant defence against invading *X.* *campestris* pv. *vesicatoria*.

On the basis of our findings, it is suggested that biochar and fly ash nanoparticles can be used for reclaiming the soil problems and enhance crop productivity depending on the nature of soil and the pathosystems under investigation.

## Data Availability

The data which is used in this finding is not available publicly due to restrictions of Punjab University Lahore, but the supporting data will be available on request.
